# Endoscopic closure of a perforated peptic ulcer using a detachable snare loop in an elderly patient at high risk for surgery

**DOI:** 10.1055/a-2906-8258

**Published:** 2026-07-13

**Authors:** Sang Soo Eom, Seokin Kang, Nam-Hoon Kim, Jong Wook Kim

**Affiliations:** 1 Department of SurgeryInje University Ilsan Paik Hospital, Inje University College of MedicineGoyangKorea (the Republic of); 2 Division of Gastroenterology, Department of Internal MedicineInje University Ilsan Paik Hospital, Inje University College of MedicineGoyangKorea (the Republic of)

Peptic ulcer perforation is a life-threatening condition and frequently progresses to sepsis; surgery remains the gold-standard treatment. 1​ Endoscopic closure of gastrointestinal perforations using detachable snare loops has been previously described 2​ ; however, these studies have predominantly addressed iatrogenic perforations, such as those following endoscopic procedures, rather than spontaneous peptic ulcer perforations. We report the case of an elderly patient at high operative risk who presented with peptic ulcer perforation and a large intra-abdominal abscess, which was successfully treated with endoscopic and radiological intervention without surgery.

**Fig. 1:**
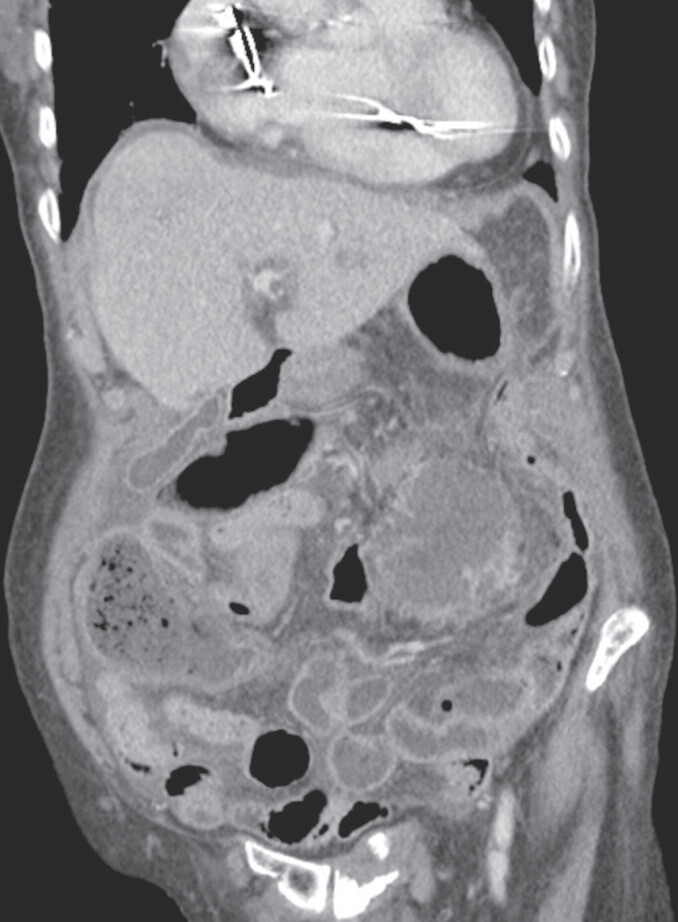
Abdominal computed tomography demonstrating a perforated gastric ulcer with a large intra-abdominal abscess measuring 7.5 cm.

A 90-year-old woman visited our hospital for abdominal pain. Computed tomography demonstrated a perforated peptic ulcer in the gastric antrum, localized peritonitis, and an intra-abdominal abscess measuring approximately 7.5 cm ( Fig. 1 ). Her medical history was notable for percutaneous coronary intervention for myocardial infarction 13 years previously and pacemaker implantation for second-degree atrioventricular block 2 years previously, and she had been on long-term aspirin therapy. Her performance status was poor (ECOG 3). Given that she was hemodynamically stable, the peritonitis was localized without evidence of free intraperitoneal air, and she was considered at high surgical risk, a nonsurgical approach was pursued.

**Fig. 2:**
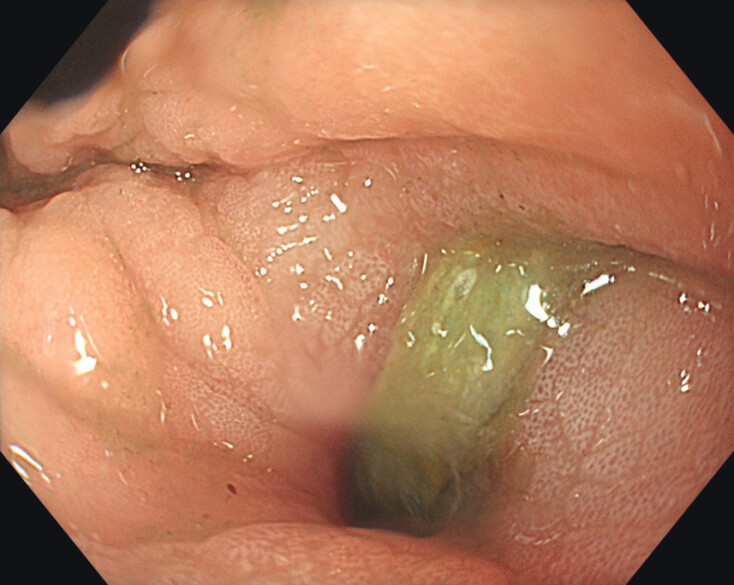
A perforated ulcer on the greater curvature of the gastric antrum with purulent discharge and edematous mucosal changes.

**Fig. 3:**
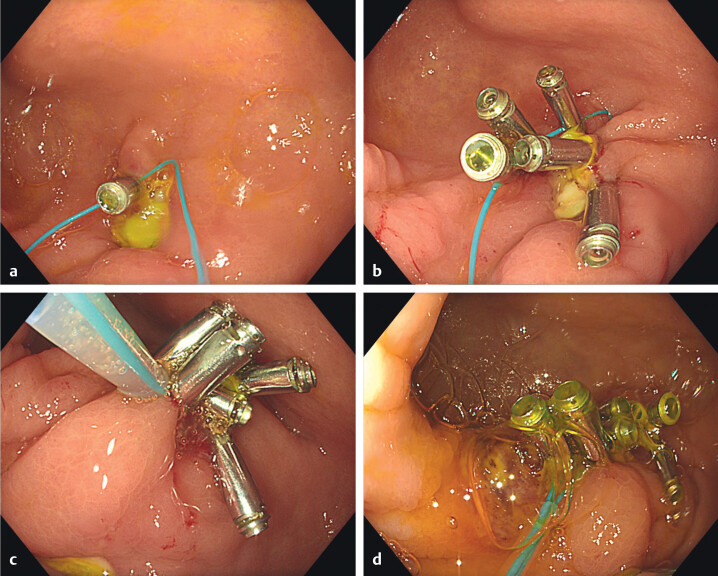
Endoscopic closure of a perforated ulcer, using a detachable snare loop and clips. ( **a** ) A detachable snare loop was anchored with a clip to the distal edge of the ulcer. ( **b** ) The detachable snare loop was fixed with more clips on the margins of the ulcer. ( **c** ) The detachable snare loop was fastened. ( **d** ) The closure of a perforated ulcer was completed.

Initially, the abscess was drained percutaneously via catheter placement. Two days later, esophagogastroduodenoscopy confirmed a 2-cm perforated ulcer on the greater curvature of the gastric antrum ( Fig. 2 ). Immediate endoscopic closure was deemed challenging because of marked mucosal edema. On day 7, endoscopic closure was successfully performed using a detachable snare loop anchored by multiple clips in a purse-string suture fashion ( Fig. 3 and Video 1 ). Follow-up computed tomography at 2 weeks demonstrated resolution of the abscess. The patient was discharged without surgical intervention.

**Video 1** Endoscopic closure of a perforated peptic ulcer using a detachable snare loop and clips in a purse-string suture fashion in a 90-year-old woman with high operative risk.


For endoscopic closure, the over-the-scope (OTS) clip 3​ and the MANTIS clip 4​ may be considered. However, the OTS clip has limited efficacy for large defects, particularly in spontaneous peptic ulcer perforation; Wei et al reported that the OTS clip was effective for the perforated peptic ulcer smaller than 1.5 cm. 5​ The larger diameter of the OTS clip system may also complicate esophageal intubation. In our 90-year-old patient, the perforation measured 2 cm, and esophageal intubation was challenging even with a standard endoscope. Evidence for the MANTIS clip in spontaneous ulcer perforation remains limited, and the periulcer inflammation and tissue friability would have hindered adequate margin approximation.

Although surgery remains the standard treatment for peptic ulcer perforation, this case suggests that endoscopic closure using a detachable snare loop and clips may serve as an alternative in elderly patients at high operative risk.

Endoscopy_UCTN_Code_TTT_1AO_2AO
